# Bone marrow metastasis in primary bronchial mucoepidermoid carcinoma: a case report

**DOI:** 10.1186/1477-7819-12-158

**Published:** 2014-05-21

**Authors:** Zhenyu Pan, Guozi Yang, Limei Qu, Tingting Yuan, Zhonghua Du, Lihua Dong

**Affiliations:** 1 Department of Radiotherapy, Norman Bethune First Hospital, Jilin University, 71 Xinmin Street, Changchun 130021, China; 2 Department of Pathology, Norman Bethune First Hospital, Jilin University, 71 Xinmin Street, Changchun 130021, China; 3 Department of Radiology, Norman Bethune First Hospital, Jilin University, 71 Xinmin Street, Changchun 130021, China; 4 Cancer Center, Norman Bethune First Hospital, Jilin University, 71 Xinmin Street, Changchun 130021, China

**Keywords:** Mucoepidermoid carcinoma, Lung, Bone marrow metastasis

## Abstract

Primary bronchial mucoepidermoid carcinoma in the lung is relatively rare. It rarely presents with the highly malignant biological characteristic of bone marrow metastasis. We describe a case of this disease with bone marrow metastasis. A 56-year-old man with the primary manifestation of bone pain and bloodstained sputum had two abnormal shadows on the left inferior lobar bronchus and peripheral tissue of the lower lobe of the left lung, respectively. Computed tomography-guided percutaneous puncture biopsy and bone imaging confirmed the diagnosis of high-grade bronchial mucoepidermoid carcinoma with bone metastasis. However, the patient soon presented with progressive hemoglobin and platelet decline and severe multi-organ hemorrhage. Subsequently, we performed bone marrow aspiration and biopsy, which revealed malignant cells and necrosis. The patient deteriorated rapidly from the disease, and died on the 16th day of admission. We hope that this case report will increase awareness of the possibility of primary high-grade bronchial mucoepidermoid carcinoma metastasizing to the bone marrow, which might be a poor prognostic factor.

## Background

Primary bronchial mucoepidermoid carcinoma, a low-malignant potential tumor of bronchial gland origin, is relatively rare and comprises approximately 0.1% of all malignant lung tumors [1]. Compared with most other lung cancers, it occurs in relatively young people [2-4]. Although mucoepidermoid carcinomas are malignant tumors, they are usually indolent, with chronic progression. Surgical treatment yields a favorable prognosis; the 5-year survival rate is 95% and adjuvant treatment is considered unnecessary. Mucoepidermoid carcinomas rarely present highly malignant biological characteristics, especially bone marrow metastasis. We report a case of unusually aggressive bronchial mucoepidermoid carcinoma with bone marrow metastasis with the aim of raising awareness of the malignant biological behavior of this tumor.

## Case presentation

**Figure 1:**
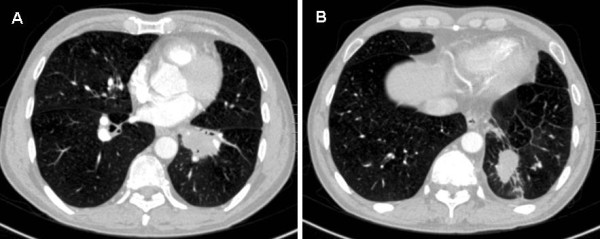
Enhanced CT scan revealing two lobulated masses measuring 30 to 40 mm in diameter in the left inferior lobar bronchus (A) and peripheral tissue of the lower lobe of the left lung (B), respectively.

**Figure 2:**
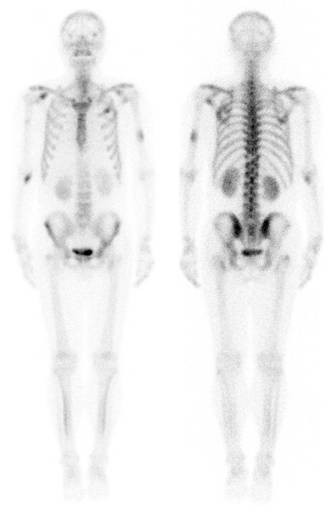
Technetium-99 m methylene diphosphonate bone scintigraphy showing metastatic involvement of the thoracic vertebra, humeri, and ribs.

A 56-year-old man presented to our hospital complaining of osphyalgia, dorsalgia, and melosalgia for 2 months, and bloodstained sputum for 2 weeks. He had been a drinker and smoker for more than 30 years, but denied personal or family history of cancer. Physical examination on admission disclosed vertebral tenderness. Routine blood examination revealed slightly decreased hemoglobin (HGB, 10.5 g/dL) and platelets (PLT, 87,000/mm^3^). On chest computed tomography (CT), we observed two lobulated masses measuring 30 to 40 mm in diameter in the left inferior lobar bronchus (Figure 1A) and peripheral tissue of the lower lobe of the left lung (Figure 1B), respectively. We also observed left hilar lymph nodes enlargement. Magnetic resonance imaging confirmed multiple sites of bone destruction of the lumbar spine, and bone scans revealed systemic multiple abnormal hypermetabolic lesions (Figure 2). Based on the clinical and auxiliary examination findings, the presumptive diagnosis was lung cancer with multiple bone metastases.

**Figure 3:**
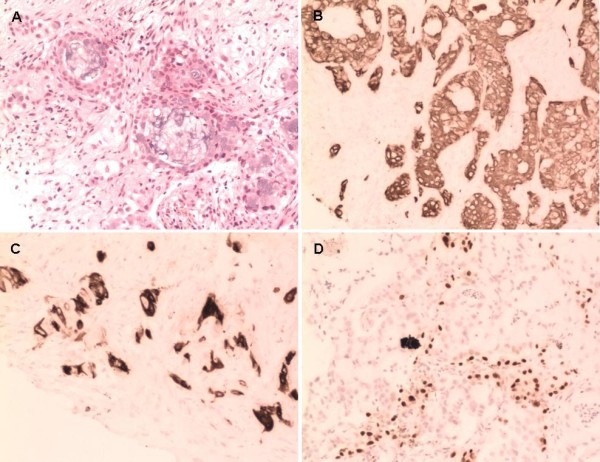
**Histological findings from lung biopsy showing high-grade bronchial mucoepidermoid carcinoma with mucin-secreting cells and intermediate cells (A: hematoxylin-eosin (HE) stain, ×200).** The tumor cells were positive for CK7, CK5/6, and TTF-1 (**B**-**D**: ×200).

On his third day in hospital, the patient underwent CT-guided percutaneous puncture biopsy of the lung and received palliative radiotherapy for the areas with severe bone destruction. The pathological findings of the lung biopsy were poorly differentiated mucoepidermoid carcinoma (Figure 3A). Ki-67 expression was about 70%. Immunohistochemical examination revealed tumor cells were positive for cytokeratin (CK) 7, CK5/6, and thyroid transcription factor-1 (TTF-1) (Figure 3B-D), which allowed us to determine that his condition was primary lung cancer. On the sixth day of admission, he presented with mild hemoptysis; routine blood examination revealed clearly decreased HGB and PLT, which were 6.2 g/dL and 56,000/mm^3^, respectively. As we suspected bone marrow infiltration by the cancer cells, we stopped radiotherapy and suggested that he undergo bone marrow aspiration and biopsy. However, he continued to deteriorate. Eight days later, we repeated the laboratory tests; Table 1 lists the results. We did not find skin petechiae or ecchymosis, or other evidence of bleeding. To carry out further examinations and prevent the risk of severe bleeding, we started supportive treatment comprising drug hemostasis and transfusion of platelet, fresh frozen plasma, and red blood cell suspension. However, the patient presented with upper gastrointestinal bleeding without any obvious cause the following day, which we suspected was stress ulceration bleeding. Thus, we administered gastrointestinal decompression and gastric acid inhibition.

**Table 1:** Laboratory test results

**Parameter**	**At admission**	**6 days after admission**	**8 days after admission**
WBC	11,700/μL	9,900/μL	9,900/μL
RBC	3,470,000/mm^3^	2,020,000/mm^3^	1,340,000/mm^3^
HGB	10.5 g/dL	6.2 g/dL	4.2 g/dL
PLT	87,000/mm^3^	56,000/mm^3^	30,000/mm^3^
PT	ND	13.2 s	19.7 s
APTT	ND	24.8 s	52.5 s
Fibrinogen	ND	1.0 g/L	0.56 g/L
D-dimer	ND	ND	0.457 mg/L
Coombs (DAT)	ND	ND	Negative

*APTT*, activated partial thromboplastin time; *DAT*, direct antibody test; *HGB*, hemoglobin; *ND*, not done; *PLT*, platelet count; *PT*, prothrombin time; *RBC*, red blood cell; *WBC*, white blood cell.

**Figure 4:**
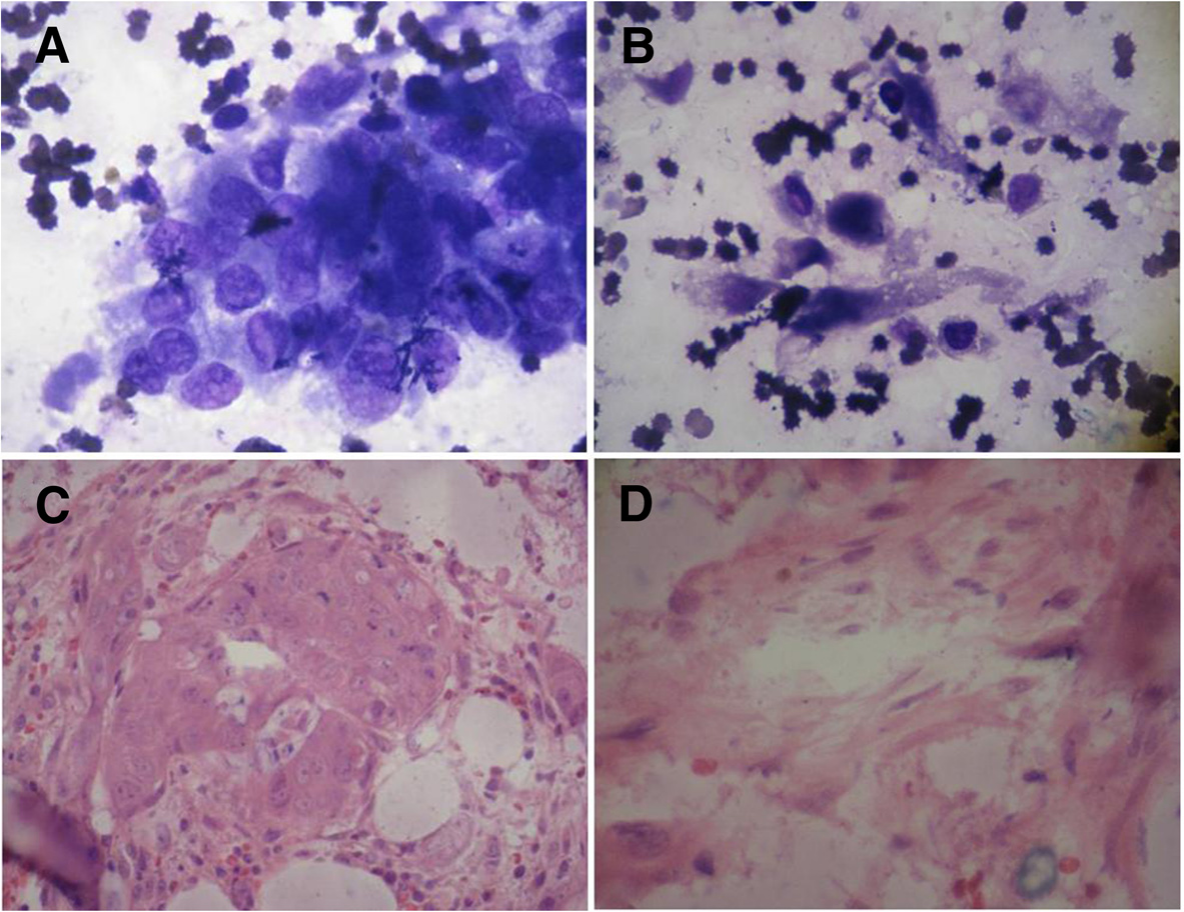
Bone marrow smear (A, B: Wright-Giemsa stain, ×1,000) and biopsy (C, D: HE stain, ×400) showing metastatic tumor cells and necrosis.

Eleven days later, his PLT count rose to 63,000/mm^3^. Bone marrow aspiration and biopsy revealed malignant cells and necrosis (Figure 4A-D). We informed the patient of the diagnosis of bone marrow metastasis with bronchial mucoepidermoid carcinoma. Unfortunately, on the day he was scheduled to receive systemic chemotherapy, the patient presented with severe respiratory tract hemorrhage. Following rapid deterioration from the disease, he died on the 16th day of admission.

## Discussion

Based on its histopathological features, we diagnosed the present lung tumor as primary high-grade bronchial mucoepidermoid carcinoma, which is relatively rare in the lung. Histologically, it is believed that mucoepidermoid carcinomas are derived from the serous and mucus glands of the trachea and bronchi. They are classified as low or high grade according to histological appearance, cellular atypia, mitotic activity, local invasion, and necrosis. It is believed that the biological behavior is associated with differentiation [5]. The prognosis of low-grade mucoepidermoid carcinomas is much better. Only very few cases of high-grade tumors with the malignant features of rapid deterioration and early distant metastases have extremely poor prognosis [6].

Bronchial mucoepidermoid carcinoma always occurs in the central bronchi. It appears on CT as an isolated, well-defined oval or lobulated mass with smooth margins arising within the bronchus [7]. It may be associated with obstructive pneumonia and atelectasis, or in a few cases, with cavitation and calcification. On CT imaging, the majority of these tumors exhibits moderate to marked enhancement. However, the present tumor was mainly located in the left inferior lobar bronchus and associated with an oval, spiculated mass in the peripheral lung tissue, a multicenter origin considered very rare.

Poorly differentiated bronchial mucoepidermoid carcinoma may present with the highly malignant biological characteristics of regional lymph node metastases. However, distant metastases, especially bone marrow metastasis, are extremely rare, and have not been reported in the available literature. Bone marrow metastasis occurs when cancer cells from a non-hematological tumor infiltrate the bone marrow via hematogenous spread or direct extension from contiguous tumor deposits [8]. Malignant cells in bone marrow smears and biopsy are a key diagnostic feature. Patients typically present with anemia, bone pain, fatigue, and progressive deterioration. Changes in the peripheral blood are always obvious decreased HGB and PLT, but normal or increased leukocytes [8].

Metastatic carcinoma in bone marrow in the exhaustion phase is a rare pathophysiological form of bone marrow metastasis. Featuring rapid onset, progressive anemia, and thrombocytopenia accompanied by severe hemorrhage, infection, and even disseminated intravascular coagulation (DIC), it is considered a lethal complication of malignant tumor [8-10]. Due to the poor performance status, systemic chemotherapy is often considered a relative contraindication for such patients. Thus, the duration of survival maintained by only supportive treatment is usually very limited [10].

The diagnosis of bone marrow metastasis in our patient was based on bone marrow smear and biopsy. The abnormal laboratory test parameters (prolonged prothrombin time and activated partial thromboplastin time, decreased fibrinogen, elevated D-dimer level) supported the diagnosis of DIC. All clinical features and auxiliary examinations were consistent with bone marrow failure induced by metastatic carcinoma. Regrettably, we were unable to perform the bone marrow examination in time and administer salvage chemotherapy to this patient in view of his personal wishes and rapid deterioration from the disease.

## Conclusions

This case demonstrates the ability of primary high-grade bronchial mucoepidermoid carcinoma to metastasize to the bone marrow, which, as has been the case in many other tumors, might be a poor prognostic factor in mucoepidermoid carcinoma. Thus, we suggest considering the probability of bone marrow metastasis if there is progressive PLT and HGB decline without any obvious reason. Bone marrow aspiration and biopsy should be performed to confirm diagnosis as soon as possible. Regardless, timely salvage chemotherapy may prolong survival and improve the prognosis.

## Consent

We obtained written informed consent from the next of kin of the patient for publication of this case report and any accompanying images. A copy of the written consent is available for review by the Editor-in-Chief of this journal.

## Competing interests

The authors declare that they have no competing interests.

## Authors’ contributions

ZYP, GZY, and LHD contributed equally to this work; participated in the care of the patient, data collection, and literature search; and drafted the manuscript. LMQ, TTY, and ZHD reviewed the CT images and photographed the bone marrow pathology. All authors participated in the conception and design of the study. ZYP and GZY wrote the first draft of the manuscript. All authors read and approved the final manuscript.

## Authors’ information

Zhenyu Pan and Guozi Yang are co-first authors.
